# Cascading effects of predator activity on tick-borne disease risk

**DOI:** 10.1098/rspb.2017.0453

**Published:** 2017-07-19

**Authors:** Tim R. Hofmeester, Patrick A. Jansen, Hendrikus J. Wijnen, Elena C. Coipan, Manoj Fonville, Herbert H. T. Prins, Hein Sprong, Sipke E. van Wieren

**Affiliations:** 1 Resource Ecology Group, Wageningen University and Research, Droevendaalsesteeg 3a, 6708 PB Wageningen, The Netherlands; 2 Center for Tropical Forest Science, Smithsonian Tropical Research Institute, Balboa, Ancon, Republic of Panamá; 3 Adaptation Physiology Group, Wageningen University and Research, De Elst 1, 6708 WD Wageningen, The Netherlands; 4 Centre for Infectious Disease Control Netherlands, National Institute for Public Health and Environment, Antonie van Leeuwenhoeklaan 9, 3721 MA Bilthoven, The Netherlands

**Keywords:** *Borrelia burgdorferi* s.l., *Borrelia miyamotoi*, carnivores, *Ixodes ricinus*, *Candidatus* Neoehrlichia mikurensis, rodents

## Abstract

Predators and competitors of vertebrates can in theory reduce the density of infected nymphs (DIN)—an often-used measure of tick-borne disease risk—by lowering the density of reservoir-competent hosts and/or the tick burden on reservoir-competent hosts. We investigated this possible indirect effect of predators by comparing data from 20 forest plots across the Netherlands that varied in predator abundance. In each plot, we measured the density of questing *Ixodes ricinus* nymphs (DON), DIN for three pathogens, rodent density, the tick burden on rodents and the activity of mammalian predators. We analysed whether rodent density and tick burden on rodents were correlated with predator activity, and how rodent density and tick burden predicted DON and DIN for the three pathogens. We found that larval burden on two rodent species decreased with activity of two predator species, while DON and DIN for all three pathogens increased with larval burden on rodents, as predicted. Path analyses supported an indirect negative correlation of activity of both predator species with DON and DIN. Our results suggest that predators can indeed lower the number of ticks feeding on reservoir-competent hosts, which implies that changes in predator abundance may have cascading effects on tick-borne disease risk.

## 1. Introduction

The incidence of zoonotic vector-borne diseases has increased in recent decades [1]. In northwestern Europe and northeastern North America, many of these are caused by pathogens that are transmitted by ticks from the *Ixodes ricinus* complex: *I. ricinus* in Europe and *Ixodes scapularis* in North America [1,2]. Both species are three-host ticks that, in temperate climates, mainly feed on small rodents or birds as larvae, on multiple host species as nymphs and on deer as adults [2]. While feeding on these hosts, ticks can become infected with pathogens that are transmitted by the host [3]. Infection prevalence in ticks often increases with the number of blood meals; hence, larvae tend to have a lower infection prevalence than nymphs, and nymphs tend to have a lower infection prevalence than adults [4]. Population densities of ticks show an opposite pattern, larvae being more abundant than nymphs, which in turn are more abundant than adults [3]. Therefore, the density of infected nymphs (DIN) is often referred to as the most important ecological parameter that, together with the level of human exposure to ticks, determines tick-borne disease risk [5].

There are large differences between areas in the incidence of tick-borne diseases [6,7]. These differences are partly caused by differences in DIN, which have been attributed to differences in climate and habitat characteristics that influence tick survival [3] and to differences in host availability, which influences tick densities and infection with pathogens [8]. DIN is often estimated as the product of the density of nymphs (DON) and nymphal infection prevalence (NIP), and depends on the absolute number of larvae that get infected while feeding on reservoir-competent hosts [8,9]. This number is determined by: (i) the abundance of reservoir-competent hosts, (ii) the average number of larvae that feed on each host individual (larval burden), and (iii) the percentage of larvae that get infected while feeding on a reservoir-competent host (realized reservoir competence). The realized reservoir competence of a host species is dependent on many factors, including the infection prevalence of the host, which is again dependent on tick burden [10,11]. Therefore, DIN is mainly determined by the density of reservoir-competent hosts and their tick burden [12].

There are reasons to assume that host density and tick burden on hosts may be influenced by predators and competitors of hosts, in at least two ways [12]. First, Ostfeld & Holt [13] reasoned that predators can reduce disease transmission by lowering the density of reservoir-competent hosts. This idea was supported by a study of tick-borne pathogens in the northeastern USA, in which the incidence of Lyme borreliosis was negatively correlated with the density of red fox (*Vulpes vulpes*) [7]. To explain these patterns, Levi *et al.* [7] provided a theoretical model in which foxes decreased the density of white-footed mice (*Peromyscus leucopus*)—the most important reservoir-competent host for *Borrelia burgdorferi*, the bacteria causing Lyme borreliosis, in North America—which then led to a decrease in DIN. However, empirical data on rodent densities and DIN in this relationship were lacking.

Second, predators might reduce DIN via non-lethal effects on prey. For example, many prey species show decreased movement and increased refuging behaviour in the presence of a predator or cues of predator presence such as predator scent [14]. As movement is an important parameter determining the encounter rate of hosts with ticks, and thus tick burden [15], predators might lower disease risk by reducing tick burden on prey species. The negative correlation between fox density and Lyme borreliosis incidence in Levi *et al.* [7] could thus be a result of a direct effect (predation) and/or an indirect effect (changed behaviour) on white-footed mice. Changes in the presence or abundance of predators could thus have cascading effects on DIN by affecting both the density of reservoir-competent hosts and the tick burden on reservoir-competent hosts.

In this study, we empirically tested for an indirect negative correlation between the abundance of mammalian predators of rodents and DIN for three tick-borne pathogens, via rodent density and tick burden on rodents. We used a study system including two rodent species—bank vole (*Myodes glareolus*) and wood mouse (*Apodemus sylvaticus*)—and three tick-borne pathogens for which these two species are the most important reservoir-competent hosts in Europe [10,16]—*Borrelia afzelii* (one of the genospecies of *B. burgdorferi* s.l.), *Borrelia miyamotoi* and *Candidatus* Neoehrlichia mikurensis. To examine empirical evidence for a cascading effect of predator activity on DIN, we first explored the relationship between predator activity, rodent density and tick burden on rodents in 20 forest plots with differing fauna in the Netherlands. Second, we explored the relationships of rodent density and tick burden on rodents with DON and DIN for the three pathogens in the same plots. We included an analysis of DON as rodents are the most important hosts feeding *I. ricinus* larvae in temperate Europe, suggesting that predators might also have a cascading effect on nymphal densities [10]. As the tick burden on rodents might be dependent on rodent density and the number of ticks in the environment [17], we also included these parameters in the analyses of tick burden on rodents. Finally, we used path analysis to determine whether there was support for an indirect correlation between predator activity and both DON and DIN.

## 2. Material and methods

### (a) Study sites

We collected data in 20 forest plots of 1 ha located within 19 forest sites in the Netherlands, with more than 5 km between sites. Sites were selected to form a large gradient in predator abundance based on distribution maps and information from the managers of the nature reserves. We assigned each plot to one of five vegetation types, based on the dominant herbaceous species (electronic supplementary material, table S1). We sampled 11 plots in 2013, and nine in 2014 (electronic supplementary material, table S1). In one site, Enkhout, we collected data in two plots 150 m apart, of which one was inside an exclosure of 3 ha. The exclosure was built to exclude large herbivores 3 years before field collection, and used by us to mimic a situation in which all larger predators were absent, which we verified with camera trapping data.

### (b) Predator activity

Rodents are known to change their behaviour in response to the presence of predators or predator scent [14,18]. The likelihood that a rodent perceives a predator increases with the amount of predators passing its home range, which is determined by the local density and activity of predators in a plot [19]. This combination of local density and activity of predators can be measured using the passage rate: a photographic capture rate corrected for differences in detectability between species and habitats [20,21].

We measured passage rates of predators using camera traps (HC500; Reconyx Inc., Holmen, WI, USA) during March–November, the period in which *I. ricinus* is most active in the Netherlands [22]. We used the camera trap set-up described by Hofmeester *et al.* [21] to obtain 18 camera positions, totalling 504 camera trapping days per plot. Theft and camera malfunction caused some variation in the total number of camera trapping days per plot (electronic supplementary material, table S1). To quantify effective detection distance (EDD), we placed a line of markers at distance intervals of 2.5 m in the centre of the view of each camera [21]. Then, for all animals that crossed the line of markers, we recorded the species and distance intervals. The frequency distribution of intervals was then used to estimate the EDD for each species per vegetation type (electronic supplementary material, table S2) [21].

The EDD estimates were used to determine passage rates per species per camera location as:2.1
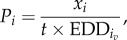
where *P_i_* is the passage rate of species *i* (in m^−1^ d^−1^), *x_i_* the number of passages of species *i*, *t* the total time the camera was active (in days) and 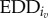 the effective detection distance of species *i* in vegetation type *v* (in metres). This passage rate is an index of local activity, described as the number of animals passing a line in front of the camera per day standardized for differences in detectability. For each plot, we calculated the plot-specific passage rate per species as the arithmetic mean of the passage rates for all deployments. Henceforth, we refer to passage rates as ‘activity’.

### (c) Rodent density and tick burden

We quantified tick burden as: (i) *larval burden*, the mean number of larvae found on individual rodents at first capture per species per plot, and as (ii) *nymphal burden*, the mean number of nymphs found on individual rodents at first capture per species per plot. We used these measures to distinguish between the chance that individual rodents become infected by feeding an infected nymph (nymphal burden) and the chance that uninfected larvae become infected by feeding on an infected rodent (larval burden). We studied the two most important rodent species feeding *I. ricinus* in the Netherlands, bank vole (*M. glareolus*) and wood mouse (*A. sylvaticus*) [23].

Rodent density and tick burden were quantified by live trapping and screening of rodents. In each plot, we established a grid of 8 × 8 (64) longworth small-mammal live traps (Heslinga Traps, Groningen, The Netherlands) with a 12 m inter-trap distance for one week in July or August, at the peak of larval activity in the Netherlands [22]. We baited the traps with maize, wheat, mealworms and a piece of carrot, and added hay as insulating material. We pre-baited the live traps for 3 days, and then checked the traps during six consecutive trapping sessions at 12 h intervals. Captured rodents were transferred from the trap into a transparent plastic bag, from which we identified the animals to species. We handled the mice and voles with care, by holding them by the scruff of their neck and counted all the ticks on the head, ears, throat and neck of the animal. We collected a stratified random selection of ticks from rodents from each plot—approximately 10% of the counted ticks—for identification to species level in the laboratory using an established identification key [24]. All collected ticks were identified as *I. ricinus*. All rodents were individually marked by clipping some of the top fur in a unique pattern, for individual identification when recaptured [25].

We estimated the density of each rodent species using the capture–mark–recapture models for closed populations presented by Otis *et al.* [26] as implemented in MARK [27], assuming that the probability of capture (*p*) and the probability of recapture (*c*) were equal and constant during trapping sessions. As we sampled 1 ha plots, we report the abundance estimates from MARK as densities per hectare. For the six combinations of plot and species where the minimum number of animals caught per species was too low to estimate a density using MARK, we used the minimum number of individuals known alive as the density estimate.

### (d) Tick density

We determined density of *I. ricinus* larvae and nymphs by collecting ticks six times in each plot, once every four weeks from April to September. Tick density was determined by blanket-dragging of 20 transects of 10 m with a 1 m^2^ cotton cloth [28] during each four-week interval, totalling 1200 m^2^ per plot. We only sampled ticks in optimal conditions: on dry days, with air temperature greater than 10°C [3], and in dry vegetation less than 60 cm high [29]. We measured air temperature and relative humidity at the start and the end of each sampling session within the vegetation using a hygro-thermometer (TH-1; Amprobe^®^, Everett, WA, USA) as both can influence the efficiency of drag sampling, resulting in biased density estimates [30]. During all sessions, dragging was performed within 5 days in all plots to minimize variation in weather conditions. We calculated the average number of nymphs per 100 m^2^ over the whole period to analyse differences between plots. All *I. ricinus* nymphs were collected in Eppendorf tubes and stored at –20°C until pathogen analysis.

To estimate larval density at the time of rodent trapping, we averaged the number of larvae dragged in July and August to estimate larval density per 100 m^2^. We used this estimate of larval density in our analysis of larval burden on the two rodent species.

### (e) Density of infected nymphs

To determine the DIN with tick-borne pathogens transmitted by rodents, we determined pathogen prevalence in all individual nymphs by qPCR using the methods described in Heylen *et al.* [31] (*B. miyamotoi*) and Jahfari *et al.* [32] (*Ca.* Neoehrlichia mikurensis). There is no qPCR available for *B. afzelii* (one of the genospecies of *B. burgdorferi* s.l.), so we used a qPCR for *B. burgdorferi* s.l. as described in Heylen *et al.* [31] followed by a conventional PCR targeting the variable 5S-23S intergenic spacer region on the positive samples of the qPCR according to the protocol described in Coipan *et al.* [33]. We determined the number of nymphs found with a co-infection of two pathogens and co-infection with all three pathogens, and estimated the overall prevalence of these co-infections using the measured prevalences.

Only 44% of the *B. burgdorferi* s.l. positive nymphs (as determined by qPCR) yielded a successful conventional PCR and sequence result. To be able to use the more sensitive qPCR results to obtain as good an estimate as possible for the infection prevalence with *B. afzelii*, we assumed that all genospecies of *B. burgdorferi* s.l. had an equal probability of being successfully sequenced. By doing so, we could approximate the infection prevalence of nymphs with *B. afzelii* for each plot as:2.2
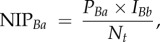
where NIP*_Ba_* is the nymphal infection prevalence with *B. afzelii*, *P_Ba_* the proportion of successful sequences identified as *B. afzelii*, *I_Bb_* the total number of nymphs infected with *B. burgdorferi* s.l. as determined by qPCR and *N_t_* the total number of nymphs tested in a plot.

We used the estimated infection prevalence with *B. afzelii* and the infection prevalence as determined by qPCR for *B. miyamotoi* and *Ca.* N. mikurensis to estimate the density of questing nymphs infected by these pathogens as:2.3
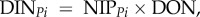
where DIN*_Pi_* is the density of questing nymphs infected with pathogen species *i* (per 100 m^2^), NIP*_Pi_* the infection prevalence in questing nymphs with pathogen species *i* and DON the density of questing nymphs as determined by blanket-dragging (per 100 m^2^).

### (f) Statistical analysis

Statistical analyses were performed in R v. 3.2.3 [34] using the *nlme* package [35] to fit linear mixed models (LMMs) and the *glmmADMB* package [36] to fit generalized LMMs (GLMMs). We first explored the data by testing for correlations between: (i) bank vole or wood mouse density, (ii) larval burden on bank voles or wood mice, or (iii) nymphal burden on bank voles or wood mice (GLMMs with negative binomial distribution and log link) and the activity of the different predator species that we detected in our plots using the *dredge* function in the *MuMIn* package [37]. Second, we explored correlations between rodent density, larval burden and nymphal burden (per species) with the density of questing nymphs (DON) and the DIN for all three pathogens using GLMMs with a negative binomial distribution and log link. We did not include nymphal burden in the analyses for wood mice as nymphal burden was correlated with larval burden in this species (GLMM: *β* = 0.63, *p* = 0.04). As temperature and humidity were correlated (LMM: *β* = −2.17, *p* = 0.001), we only added humidity as a covariate in all models of DON and DIN to correct for possible effects of these parameters on questing tick activity. We tested for collinearity by calculating the variance inflation factor [38], which was below 2 for all reported models. We allowed a random intercept per vegetation type nested within year in all models including DON or DIN to correct for possible differences in drag-sampling efficiency between vegetation types and possible differences in questing tick densities between years owing to differences in weather conditions. We allowed a random intercept per year for all other models to correct for possible differences between years.

We used confirmatory path analysis using directional separation [39], to quantify an indirect correlation of predator activity with DON and DIN via rodent density or larval burden on rodents. When data used in a path analysis have a hierarchical or multi-level structure, directional separation can be used to test causal models [39]. A causal model is rejected when variables that are only indirectly connected by a causal path are not independent from each other conditional on the variables that are direct causes of either of the variables to be tested. An example from our model is the relationship between predator activity and DIN, which would be independent conditional on rodent density and larval burden on rodents (independence claim) if a causal relationship exists. This independence can be statistically tested using a model in which all parameters in the independence claim are included. Using the example above that would be a model regressing DIN with predator activity, rodent density and larval burden, where predator activity should not be correlated with DIN when rodent density and larval burden are held constant.

We determined and tested the independence claims for the causal models (electronic supplementary material, S3) and calculated *C* values as described by Shipley [39] for each of the combinations of DON and DIN for the different pathogens per rodent species. We only tested path models for predators and rodent characteristics where we found significant correlations (*α* = 0.05) for the individual tests. Causal models were rejected if the *C* value was unlikely to have occurred by chance (*p* < 0.05) using a *χ*^2^-test [39]. We tested the path coefficients for not rejected causal models using GLMMs with a negative binomial distribution and a log link for all paths.

As larval densities, rodent densities, larval burdens and nymphal burdens were over-dispersed, we log_10_ transformed these parameters to approximate normality in models in which these parameters were included as explanatory variables. If the parameter included estimates of zero, we added the lowest measured positive number to circumvent problems with the transformation. We standardized all parameters, when applied after transformation, by extracting the mean and dividing by 2 s.d. [40] to obtain standardized regression coefficients.

We used a *χ*^2^-test to test for differences in observed prevalence of co-infections with the different pathogens in all questing nymphs, and expected prevalence of co-infections based on the infection prevalence of the separate pathogens, to test for associations between the pathogens.

## 3. Results

We found large variation between plots in the DIN for *B. afzelii*, *B. miyamotoi* and *Ca.* Neoehrlichia mikurensis, and also in the activity of mammalian predators of rodents (see [41] for full dataset). Red fox (*V. vulpes*) was present in most plots (18 out of 20), followed by European pine marten (*Martes martes*; 12 out of 20), stone marten (*Martes foina*; 6 out of 20) and European polecat (*Mustela putorius*; 5 out of 20). Where they were present, red fox also showed the highest activity (mean ± standard deviation) 0.0080 ± 0.0061 m^−1^ d^−1^, followed by Pine marten 0.0041 ± 0.0037 m^−1^ d^−1^, stone marten 0.0011 ± 0.0011 m^−1^ d^−1^ and polecat 0.0011 ± 0.0006 m^−1^ d^−1^.

We excluded one plot (Duin en Kruidberg) from our analyses as we did not catch any rodents in this site and thus could not determine tick burden on rodents. For the analyses involving tick burden on bank voles (*M. glareolus*), we also excluded two sites (Amsterdamse Waterleiding Duinen and Schoorlse Duinen) where we did not catch any bank voles.

**Figure 1.**
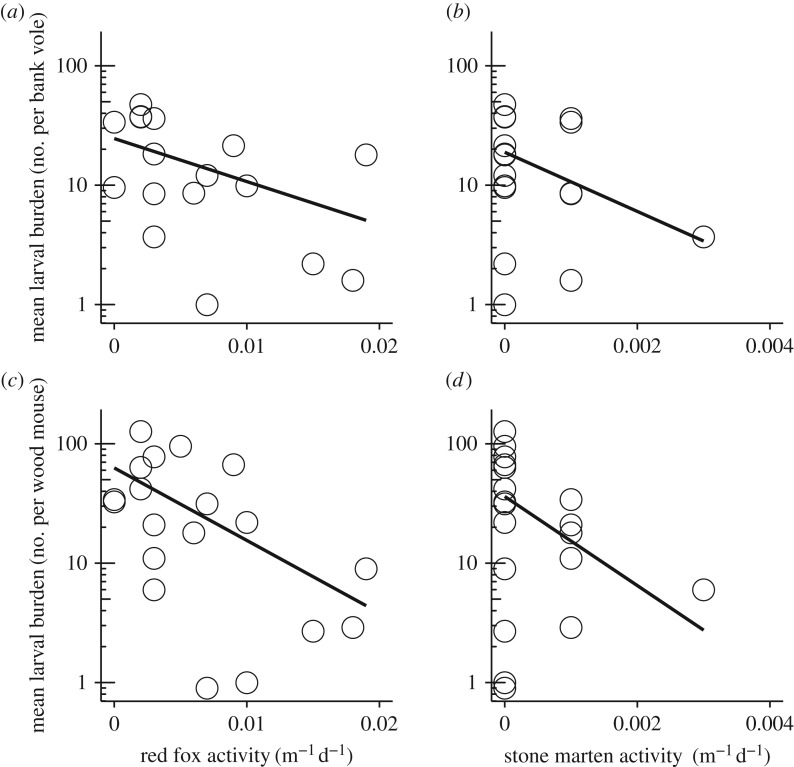
Correlations between the mean larval burden on bank voles and (*a*) red fox and (*b*) stone marten activity, and correlation between the mean larval burden on wood mice and (*c*) red fox and (*d*) stone marten activity. Points show the raw data, solid lines show the model predictions for a model including both predator species.

**Figure 2.**
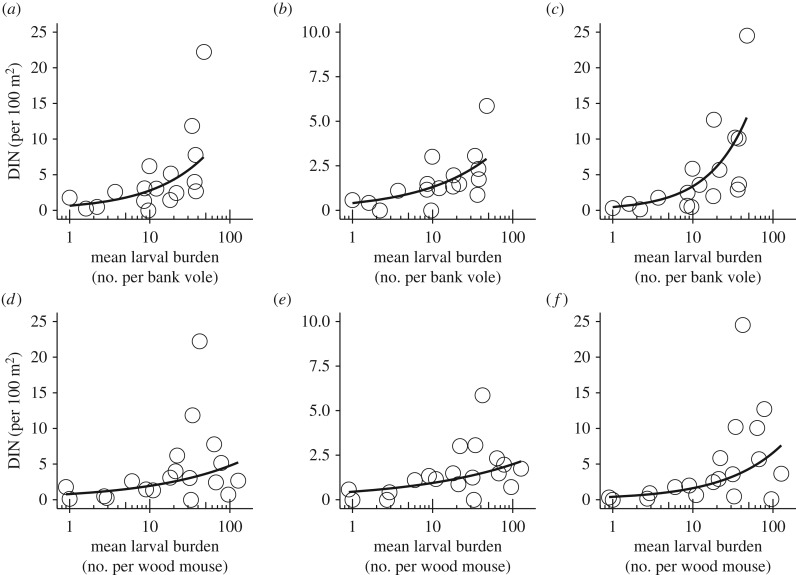
Correlations between the mean larval burden on rodents and DIN for (*a*) *B. afzelii*, (*b*) *B. miyamotoi* and (*c*) *Ca.* Neoehrlichia mikurensis for bank voles, and (*d*) *B. afzelii*, (*e*) *B. miyamotoi* and (*f*) *Ca.* N. mikurensis for wood mice. Points show the raw data, solid lines show the model predictions for full models as presented in the electronic supplementary material, S5.

Exploratory analyses showed that bank vole density increased with pine marten activity (GLMM: *β* = 1.45, *p* = 0.04) but was not correlated with the activity of any of the other predators (electronic supplementary material, S4). Wood mouse (*A. sylvaticus*) density was not correlated with the activity of any of the predators, although there was a near significant trend for polecat (*β* = 1.22, *p* = 0.06; electronic supplementary material, S4). Larval burden on bank voles (*β*_fox_ = −0.99, *p*_fox_ = 0.004; *β*_marten_ = −0.91, *p*_marten_ = 0.02) and wood mice (*β*_fox_ = −1.60, *p*_fox_ < 0.001; *β*_marten_ = −1.32, *p*_marten_ < 0.001) decreased with the activity of red fox and stone marten (figure 1; electronic supplementary material, S4). Nymphal burden on bank voles and wood mice was not correlated with predator activity (electronic supplementary material, S4), although there was a negative trend of nymphal burden on wood mice with red fox activity (*β* = −1.29, *p* = 0.11). Further exploration showed that both DON and DIN for all three pathogens increased with larval burden on both bank voles and wood mice (figure 2; electronic supplementary material, S5).

**Figure 3.**
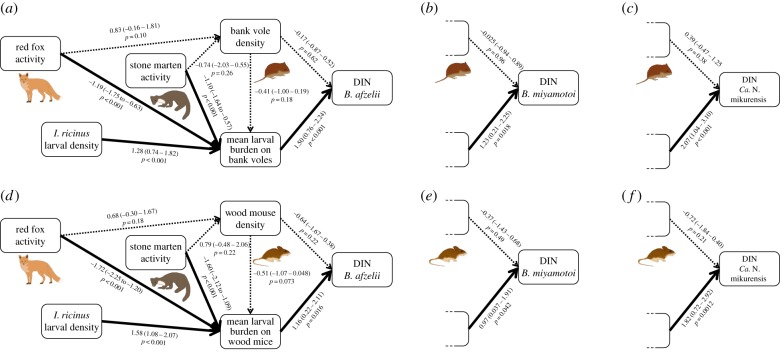
Path diagrams showing direct and indirect correlations of predator activity and larval density with rodent density, mean larval burden on rodents and DIN for (*a*) bank voles and *B. afzelii*, (*b*) bank voles and *B. miyamotoi*, (*c*) bank voles and *Ca.* Neoehrlichia mikurensis, (*d*) wood mice and *B. afzelii*, (*e*) wood mice and *B. miyamotoi*, and (*f*) wood mice and *Ca*. N. mikurensis. The left part of the diagram is identical for all models per species and therefore only shown once. Thick arrows indicate statistically supported paths. Path coefficients have 95% confidence interval between brackets. (Online version in colour.)

Path analyses supported an indirect correlation of both red fox and stone marten activity with DON (electronic supplementary material, figure S6) and DIN for *B. afzelii*, *B. miyamotoi* and *Ca.* N. mikurensis via mean larval burden on both bank voles and wood mice (figure 3). One of the independence claims for the path concerning DON was not met (electronic supplementary material, S3), so this path was added to the path diagram, after which both the path models for bank vole (*C* = 4.8, *p* = 0.60) and for wood mouse (*C* = 5.8, *p* = 0.47) were not rejected (electronic supplementary material, figure S6). None of the tested path models for DIN for bank vole (*B. afzelii*: *C* = 5.3, *p* = 0.72; *B. miyamotoi*: *C* = 3.7, *p* = 0.88; *Ca.* N. mikurensis: *C* = 3.4, *p* = 0.90) and for wood mouse (*B. afzelii*: *C* = 9.4, *p* = 0.31; *B. miyamotoi*: *C* = 7.2, *p* = 0.52; *Ca.* N. mikurensis: *C* = 7.9, *p* = 0.45) were rejected. Consistent with the previous analyses, not all path coefficients were significantly different from zero: there was no correlation of red fox and stone marten activity with either bank vole or wood mouse density, no correlation between bank vole density and mean larval burden on bank voles, no correlation between wood mouse density and mean larval burden on wood mice and no correlation between bank vole or wood mouse density and DIN for any of the pathogens (figure 3). Larval burden on bank voles and wood mice increased with larval density, and decreased with red fox and stone marten activity, as expected (figure 3). For each of the pathogens, DIN increased with larval burden on rodents also after correcting for differences in bank vole or wood mouse density (figure 3).

Among 16 617 questing nymphs that we screened, we found very low co-infection percentages with an over-representation of all four possible co-infections: *B. afzelii* and *B. miyamotoi* (observed % = 0.084, expected % = 0.045, *χ*^2^-value = 4.3, *p* = 0.038), *B. afzelii* and *Ca.* N. mikurensis (observed % = 0.54, expected % = 0.13, *χ*^2^-value = 168.1, *p* < 0.001), *B. miyamotoi* and *Ca.* N. mikurensis (observed % = 0.18, expected % = 0.12, *χ*^2^-value = 4.0, *p* = 0.047) and the three pathogens together (observed % = 0.023, expected % = 0.0027, *χ*^2^-value = 19.8, *p* < 0.001).

## 4. Discussion

Theory predicts that predators can reduce the DIN by suppressing reservoir-competent hosts and the encounter rate between ticks and reservoir-competent hosts [7,13]. Changes in predator presence and activity might thus have cascading effects on disease risk. We carried out a field study to determine whether variation in the density of questing nymphs (DON) and DIN was explained by variation in predator activity via both rodent density and larval burden on rodents. We found that larval burden on two important reservoir-competent hosts, bank vole (*M. glareolus*) and wood mouse (*A. sylvaticus*), decreased with activity of two predators: red fox (*V. vulpes*) and stone marten (*Martes foina*). Overall DON as well as DIN for three tick-borne pathogens—*B. afzelii*, *B. miyamotoi* and *Ca.* Neoehrlichia mikurensis—increased with larval burden on these rodents. Path analyses supported an indirect (negative) correlation between both predator species and DON, and DIN for all three pathogens, which is in agreement with theory.

Path analyses revealed an indirect negative correlation of both red fox and stone marten activity with DON and DIN for all three tick-borne pathogens via differences in the mean larval burden on rodents, even after correcting for an indirect correlation with questing larval density (figure 3; electronic supplementary material, figure S6). Red foxes and stone martens generally carry few *I. ricinus* [42–44]. Therefore, we conclude that it is unlikely that the negative correlation between predator activity and larval burden on bank voles and wood mice is caused by a dilution effect where foxes and martens divert ticks that would otherwise feed on rodents [12]. We suggest two other mechanisms that might explain the negative correlation between predator activity and larval burden on bank voles and wood mice. First, bank voles and wood mice can become less active in areas with more cues of predator presence [18], reducing their encounter rate with ticks and therefore tick burden [15]. Second, those animals that do move more and therefore acquire more ticks [15] might also have a higher risk of being predated, leading to a selective predation on highly infested animals [45]. Further research estimating the day range of rodents in relation to predation risk and tick burden in areas differing in predator activity is needed to test these hypotheses.

Our analyses focused on the determinants of DON and DIN. The reason that we did not do the same for NIP is that we lacked data on one of the main determinants: the number of larvae feeding on non-rodent hosts [9]. There is certainly a potential for predators to influence NIP, namely by changing the number of larvae feeding on rodents and possibly also the number of larvae feeding on non-rodent hosts that are prey to the same predators. An analysis of the entire host assemblage is needed to fully understand how predators influence NIP, and thereby one of the parameters determining tick-borne disease risk.

We sampled predators, rodents and questing ticks in each plot in the same year. However, where there is a time lag between these factors, we might have missed correlations. By measuring all variables in the same year, we assumed that densities were constant between years, but this assumption is probably invalid, especially for rodents [46]. Ostfeld *et al.* [47] found a positive correlation between rodent density in year *t* − 1 and the density of *I. scapularis* nymphs in year *t*. Similarly, there might be a time lag on potential effects of predators on rodent density [13]. This is further complicated by the fact that on different spatial scales (i) predator activity might reduce rodent population densities (negative correlation) [46], while at the same time, (ii) patches with high rodent density might attract predators from the surrounding area (positive correlation) [48]. We mainly found support for the second relationship as all correlations between predator activity and rodent density in our study were positive (electronic supplementary material, S4). Therefore, correlational studies on a small spatial scale (less than or equal to 1 ha) might not be able to show regulation of rodents by predators. Studies that span multiple years on several spatial scales are thus needed to better understand the correlations between predator activity and rodent and tick density.

We found a strong identity effect of red fox and stone marten compared with the other mammalian predators of rodents (electronic supplementary material, S4). All predators were generalist foragers that feed on a large variety of food items, but red fox has the highest proportion of small rodent biomass in its diet [49–51], which might explain the strong identity effect. Second, the identity effect might relate to predator size as red fox and stone marten were the two largest predators that we recorded [52]. Reducing movement and increasing refuging behaviour may be more effective as strategy to avoid a larger predator than as strategy to avoid a smaller predator that can also hunt in dense vegetation or enter rodent burrows.

We found further support for a reservoir role of bank vole and wood mouse for *B. miyamotoi* and *Ca.* N. mikurensis as co-infections of these pathogens with *B. afzelii* in questing nymphs occurred more than expected by chance. This suggests that larvae get infected with these three pathogens while feeding on the same host species, suggesting that the same host species that maintain *B. afzelii* also maintain *B. miyamotoi* and *Ca.* N. mikurensis. This is further supported by the higher standardized correlation coefficients for larval burden in correlation with DIN for these pathogens compared with the results for DON. Overall, the patterns were less strong for *B. miyamotoi*, probably because infection by larvae and transovarial transmission also play a role in the maintenance of this pathogen, but not for the others [53].

This study is, to our knowledge, the first to find empirical support for a negative correlation between the activity of predators, the density of questing nymphs and DIN for tick-borne pathogens. Our study also highlights the importance of differences in larval burden between sites as these were correlated with differences in nymphal densities and DIN between sites. The results suggest that predators can indeed lower the number of ticks feeding on reservoir-competent hosts, which implies that changes in predator abundance may have cascading effects on tick-borne disease risk. The emergence of cascading effects of predator activity on tick-borne disease risk calls for the appreciation and protection of predator species such as red fox, many of which are persecuted across Europe [54].

## Supplementary Material

Table S1

## Supplementary Material

Table S2

## Supplementary Material

Supplementary material S3

## Supplementary Material

Supplementary material S4

## Supplementary Material

Supplementary material S5

## Supplementary Material

Figure S6

## Data accessibility

The full dataset used in the analyses is available at the Dryad Data Repository: http://dx.doi.org/10.5061/dryad.th1s6 [41].
