# Supplementary material for: Cascading effects of predator activity on tick-borne disease risk
Source: Proc Biol Sci. 2017 Jul 19;284(1859):20170453. doi: 10.1098/rspb.2017.0453 (PMC5543215; doi:10.1098/rspb.2017.0453)
Supplement: Table S1 [file rspb20170453supp1.pdf]

Supplementary material belonging to the manuscript: Hofmeester, T.R., Jansen, P.A., Wijnen, H.J., Coipan, E.C., Fonville, M., Prins, H.H.T., Sprong, H., and van Wieren, S.E. Cascading effects of predator activity on tick-borne disease risk. Proceedings of the Royal Society B: Biological sciences. DOI: 10.1098/rspb.2017.0453

Table S1. Characteristics of the research sites, and sampling effort (camera trap days).

Site	Vegetation		Coordinates [b]		Year	Effort (days)
	Overstory	Undergrowth [a]	Latitude	Longitude		
Amsterdamse Waterleiding Duinen	Mixed forest	*Calamagrostis epigejos*	52°20'36''N	4°33'58''E	2014	492
Bergherbos	Mixed forest	*Deschampsia flexuosa*	51°55'14''N	6°14'30''E	2013	504
Buunderkamp	Scots pine forest	*Vaccinium myrtillus*	52°00'56''N	5°44'50''E	2013	504
Duin en Kruidberg	Mixed forest	*Calamagrostis epigejos*	52°26'16''N	4°36'18''E	2013	504
Deelerwoud	Scots pine forest	*Vaccinium myrtillus*	52°05'51''N	5°56'42''E	2014	504
Enkhout	Scots pine forest	*Vaccinium myrtillus*	52°16'25''N	5°54'49''E	2013	495 / 504[c]
Herperduin	Mixed forest	*Molinia caerulea*	51°45'33''N	5°36'53''E	2014	504
Halfmijl	Mixed forest	*Molinia caerulea*	51°25'23''N	5°19'09''E	2013	504
Kremboong	European oak forest	Fern (*Dryopteris dilatata*)	52°45'13''N	6°31'16''E	2013	504
Maashorst	Mixed forest	*Deschampsia flexuosa*	51°42'44''N	5°35'24''E	2014	504
Pettemerduin	European oak forest	Fern (*Polypodium vulgare*)	52°46'33''N	4°40'19''E	2014	499
Planken Wambuis	Scots pine forest	*Vaccinium myrtillus*	52°01'54''N	5°48'36''E	2013	441
Rheebruggen	European oak forest	Fern (*Dryopteris dilatata*)	52°46'60''N	6°17'44''E	2014	504
Schoorlse Duinen	Mixed forest	*Molinia caerulea*	52°41'47''N	4°40'01''E	2013	504
Stameren	Mixed forest	*Deschampsia flexuosa*	52°03'38''N	5°21'01''E	2014	486
Valenberg	Scots pine forest	*Vaccinium myrtillus*	52°15'33''N	5°48'47''E	2014	391
Vijverhof	Mixed forest	*Deschampsia flexuosa*	52°09'43''N	5°13'43''E	2013	507
Vledderhof	European oak forest	Fern (*Dryopteris dilatata*)	52°52'46''N	6°14'25''E	2014	504
Zwanemeerbos	European oak forest	Fern (*Pteridium aquilinum*)	53°00'46''N	6°45'19''E	2013	504

[a] The most dominant plant species in the herbaceous layer, different fern species were combined into one vegetation type.

[b] Coordinates as measured with a handheld GPS (Garmin eTrex 20) in the plot centre.

[c] The number of camera trapping days outside of the exclosure (left) and inside the exclosure (right).
