# Supplementary material for: Cascading effects of predator activity on tick-borne disease risk
Source: Proc Biol Sci. 2017 Jul 19;284(1859):20170453. doi: 10.1098/rspb.2017.0453 (PMC5543215; doi:10.1098/rspb.2017.0453)
Supplement: Table S2 [file rspb20170453supp2.pdf]

Supplementary material belonging to the manuscript: Hofmeester, T.R., Jansen, P.A., Wijnen, H.J., Coipan, E.C., Fonville, M., Prins, H.H.T., Sprong, H., and van Wieren, S.E. Cascading effects of predator activity on tick-borne disease risk. Proceedings of the Royal Society B: Biological sciences. DOI: 10.1098/rspb.2017.0453

Table S2. Estimated Effective Detection Distances (EDD), for the medium-sized to large mammal species photographed with camera traps, per habitat type.

**Species**	**Body mass[a] (kg)**	**EDD (m) by Habitat[b]**				
		CE[c]	DF[c]	FE[c]	MC[c]	VM[c]
**Eurasian red squirrel (*Sciurus vulgaris*)**	0.3	4.0	3.0	2.7	2.5	2.4
**European badger (*Meles meles*)**	11.8		5.4	5.4	5.5	4.5
**European hare (*Lepus europaeus*)**	3.8	4.9	4.5	4.3	4.3	3.7
**European hedgehog (*Erinaceus europaeus*)**	0.8	4.3		3.2	3.0	
**European pine marten (*Martes martes*)**	1.3	4.5	3.7	3.5	3.4	3.0
**European Rabbit (*Oryctolagus cuniculus*)**	1.6		3.8		3.5	
**Fallow deer (*Dama dama*)**	57.2	6.2		7.2		5.9
**Feral house cat (*Felis catus*)**	2.9		4.3	4.1	4.0	
**Polecat (*Mustela putorius*)**	1.0	4.4		3.4	3.2	
**Red deer (*Cervus elaphus*)**	240.9					7.6
**Red fox (*Vulpes vulpes*)**	4.8	5.0	4.6	4.5	4.5	3.8
**Roe deer (*Capreolus capreolus*)**	22.5	5.7	6.0	6.1	6.3	5.0
**Stone marten (*Martes foina*)**	1.7		3.9	3.7	3.6	
**Wild boar (*Sus scrofa*)**	84.5					6.3

[a] Body mass values are taken from the PanTHERIA database. Jones K.E. et al. 2009 PanTHERIA: a species-level database of life history, ecology, and geography of extant and recently extinct mammals. *Ecology* **90**(9), 2648-2648. (doi:10.1890/08-1494.1).

[b] The abbreviations are related to the dominant species of vegetation in the herbaceous layer in the 1 hectare forest plot. CE = *Calamagrostis epigejos*, DF = *Deschampsia flexuosa*, FE = fern species (*Dryopteris dilatata*, *Polypodium vulgare* or *Pteridium aquilinum*), MC = *Molinia caerulea*, and VM = *Vaccinium myrtillus*.

[c] Estimates of EDD given are based on a point model with a half-normal detection probability function with $\log_{10}$-transformed body mass as covariate.
