# Supplementary material for: Cascading effects of predator activity on tick-borne disease risk
Source: Proc Biol Sci. 2017 Jul 19;284(1859):20170453. doi: 10.1098/rspb.2017.0453 (PMC5543215; doi:10.1098/rspb.2017.0453)
Supplement: Supplementary material S3 [file rspb20170453supp3.pdf]

Supplementary material belonging to the manuscript: Hofmeester, T.R., Jansen, P.A., Wijnen, H.J., Coipan, E.C., Fonville, M., Prins, H.H.T., Sprong, H., and van Wieren, S.E. Cascading effects of predator activity on tick-borne disease risk. Proceedings of the Royal Society B: Biological sciences. DOI: 10.1098/rspb.2017.0453

**Supplementary material S3.**

Description and results of the basis set of d-separation (d-sep) independence claims testing the indirect correlation between predator activity, larval *Ixodes ricinus* density, rodent density, larval burden on rodents, the density of questing nymphs (DON) and the density of infected nymphs (DIN) for *Borrelia afzelii*, *B. miyamotoi* and *Candidatus* Neoehrlichia mikurensis. $X_1$ = Red fox (*Vulpes vulpes*) activity, $X_2$ = Stone marten (*Martes foina*) activity, $X_3$ = Larval density, $X_4$ = Rodent density, $X_5$ = Average larval burden on rodents, $X_6$ = DON, $X_7$ = DIN *B. afzelii*, $X_8$ = DIN *B. miyamotoi* and $X_9$ = DIN *Ca.* N. mikurensis; VT = Vegetation type.

Table S5.1. The basis set of d-separation (d-sep) claims for Bank vole models.

| D-sep claim of independence[a] | Mixed model[a] | Null probability (distribution) |
|---|---|---|
| $(X_3, X_4) \mid \{X_1, X_2\}$ | $X_4 \sim X_1 + X_2 + X_3 + (1\|Year/VT)$ | 0.87 (neg.bionomial) |
| $(X_1, X_6) \mid \{X_4, X_5\}$ | $X_6 \sim X_1 + X_4 + X_5 + (1\|Year/VT)$ | 0.94 (neg. binomial) |
| $(X_2, X_6) \mid \{X_4, X_5\}$ | $X_6 \sim X_2 + X_4 + X_5 + (1\|Year/VT)$ | 0.11 (neg. binomial) |
| $(X_3, X_6) \mid \{X_4, X_5\}$ | $X_6 \sim X_3 + X_4 + X_5 + (1\|Year/VT)$ | <0.001 (neg. binomial) |
| $(X_1, X_7) \mid \{X_4, X_5\}$ | $X_7 \sim X_1 + X_4 + X_5 + (1\|Year/VT)$ | 0.25 (neg. binomial) |
| $(X_2, X_7) \mid \{X_4, X_5\}$ | $X_7 \sim X_2 + X_4 + X_5 + (1\|Year/VT)$ | 0.66 (neg. binomial) |
| $(X_3, X_7) \mid \{X_4, X_5\}$ | $X_7 \sim X_3 + X_4 + X_5 + (1\|Year/VT)$ | 0.49 (neg. binomial) |
| $(X_1, X_8) \mid \{X_4, X_5\}$ | $X_8 \sim X_1 + X_4 + X_5 + (1\|Year/VT)$ | 0.97 (neg. binomial) |
| $(X_2, X_8) \mid \{X_4, X_5\}$ | $X_8 \sim X_2 + X_4 + X_5 + (1\|Year/VT)$ | 0.92 (neg. binomial) |
| $(X_3, X_8) \mid \{X_4, X_5\}$ | $X_8 \sim X_3 + X_4 + X_5 + (1\|Year/VT)$ | 0.20 (neg. binomial) |
| $(X_1, X_9) \mid \{X_4, X_5\}$ | $X_9 \sim X_1 + X_4 + X_5 + (1\|Year/VT)$ | 0.96 (neg. binomial) |
| $(X_2, X_9) \mid \{X_4, X_5\}$ | $X_9 \sim X_2 + X_4 + X_5 + (1\|Year/VT)$ | 0.99 (neg. binomial) |
| $(X_3, X_9) \mid \{X_4, X_5\}$ | $X_9 \sim X_3 + X_4 + X_5 + (1\|Year/VT)$ | 0.22 (neg. binomial) |

Table S5.2. The basis set of d-separation (d-sep) claims for Wood mouse models.

| D-sep claim of independence[a] | Mixed model[a] | Null probability (distribution) |
|---|---|---|
| $(X_3, X_4) \mid \{X_1, X_2\}$ | $X_4 \sim X_1 + X_2 + X_3 + (1\|Year/VT)$ | 0.54 (neg.bionomial) |
| $(X_1, X_6) \mid \{X_4, X_5\}$ | $X_6 \sim X_1 + X_4 + X_5 + (1\|Year/VT)$ | 0.85 (neg. binomial) |
| $(X_2, X_6) \mid \{X_4, X_5\}$ | $X_6 \sim X_2 + X_4 + X_5 + (1\|Year/VT)$ | 0.12 (neg. binomial) |
| $(X_3, X_6) \mid \{X_4, X_5\}$ | $X_6 \sim X_3 + X_4 + X_5 + (1\|Year/VT)$ | <0.001 (neg. binomial) |
| $(X_1, X_7) \mid \{X_4, X_5\}$ | $X_7 \sim X_1 + X_4 + X_5 + (1\|Year/VT)$ | 0.09 (neg. binomial) |
| $(X_2, X_7) \mid \{X_4, X_5\}$ | $X_7 \sim X_2 + X_4 + X_5 + (1\|Year/VT)$ | 0.72 (neg. binomial) |
| $(X_3, X_7) \mid \{X_4, X_5\}$ | $X_7 \sim X_3 + X_4 + X_5 + (1\|Year/VT)$ | 0.27 (neg. binomial) |
| $(X_1, X_8) \mid \{X_4, X_5\}$ | $X_8 \sim X_1 + X_4 + X_5 + (1\|Year/VT)$ | 0.59 (neg. binomial) |
| $(X_2, X_8) \mid \{X_4, X_5\}$ | $X_8 \sim X_2 + X_4 + X_5 + (1\|Year/VT)$ | 0.73 (neg. binomial) |
| $(X_3, X_8) \mid \{X_4, X_5\}$ | $X_8 \sim X_3 + X_4 + X_5 + (1\|Year/VT)$ | 0.12 (neg. binomial) |
| $(X_1, X_9) \mid \{X_4, X_5\}$ | $X_9 \sim X_1 + X_4 + X_5 + (1\|Year/VT)$ | 0.54 (neg. binomial) |
| $(X_2, X_9) \mid \{X_4, X_5\}$ | $X_9 \sim X_2 + X_4 + X_5 + (1\|Year/VT)$ | 0.83 (neg. binomial) |
| $(X_3, X_9) \mid \{X_4, X_5\}$ | $X_9 \sim X_3 + X_4 + X_5 + (1\|Year/VT)$ | 0.08 (neg. binomial) |
