# Supplementary material for: Cascading effects of predator activity on tick-borne disease risk
Source: Proc Biol Sci. 2017 Jul 19;284(1859):20170453. doi: 10.1098/rspb.2017.0453 (PMC5543215; doi:10.1098/rspb.2017.0453)
Supplement: Supplementary material S4 [file rspb20170453supp4.pdf]

Supplementary material belonging to the manuscript: Hofmeester, T.R., Jansen, P.A., Wijnen, H.J., Coipan, E.C., Fonville, M., Prins, H.H.T., Sprong, H., and van Wieren, S.E. Cascading effects of predator activity on tick-borne disease risk. Proceedings of the Royal Society B: Biological sciences. DOI: 10.1098/rspb.2017.0453

Supplementary material S4.

Full model results of exploratory analyses testing the correlations between predator activity and rodent characteristics (density, larval and nymphal burden). All models were generalized linear mixed models with a negative binomial distribution and log link function with a random intercept per year. Values given are estimates of the standardized correlation coefficient for passage rate per species. Comparisons of model fit are given by the difference in small-sample-size corrected Akaike Information Criterion (ΔAICc) and calculated model weight (ω_m). Only models with ΔAICc < 4 are shown. Significant coefficient estimates are presented in bold, * for p < 0.05, ** for p < 0.01, *** for p < 0.001.

Table S4.1. Model outcomes for the correlation between predator activity and Bank vole (*Myodes glareolus*) density.

Model	Intercept	Pine marten[a]	Polecat[b]	Red fox[c]	Stone marten[d]	ΔAICc	ω_m
1	**3.13*** **	**1.45***				0	0.351
2	**3.28*** **					0.97	0.216
3	**3.23*** **			0.65		2.80	0.086
4	**3.10*** **	1.48	0.56			3.36	0.065
5	**3.11*** **	1.53			-0.51	3.49	0.061
6	**3.11*** **	1.27		0.37		3.50	0.061
7	**3.27*** **		0.64			3.77	0.053

[a] *Martes martes*; [b] *Mustela putorius*; [c] *Vulpes vulpes*; [d] *Martes foina*

Table S4.2. Model outcomes for the correlation between predator activity and Wood mouse (*Apodemus sylvaticus*) density.

Model	Intercept	Pine marten	Polecat	Red fox	Stone marten	ΔAICc	ω_m
1	**2.51*** **		1.22			0	0.265
2	**2.60*** **					0.47	0.209
3	**2.56*** **			0.73		1.73	0.112
4	**2.56*** **				0.87	2.04	0.095
5	**2.50*** **		0.94	0.46		2.93	0.061
6	**2.49*** **		1.03		0.57	2.98	0.060
7	**2.59*** **	0.39				3.23	0.053
8	**2.51*** **	0.22	1.16			3.61	0.044
9	**2.52*** **			0.68	0.79	3.94	0.037

Table S4.3. Model outcomes for the correlation between predator activity and larval burden on Bank voles.

Model	Intercept	Pine marten	Polecat	Red fox	Stone marten	ΔAICc	ω_m
1	**2.67***			**-0.99**	**-0.91*	0	0.243
2	**2.75***			**-0.85*		0.01	0.242
3	**2.83***					0.71	0.171
4	**2.77***				-0.72	2.26	0.079
5	**2.74***		-0.52	-0.84		2.74	0.062
6	**2.82***		-0.56			2.83	0.059

Table S4.4. Model outcomes for the correlation between predator activity and larval burden on Wood mice.

Model	Intercept	Pine marten	Polecat	Red fox	Stone marten	ΔAICc	ω_m
1	**3.23***			**-1.60***	**-1.32***	0	0.606
2	**3.38***			**-1.48**		3.31	0.116

Table S4.5. Model outcomes for the correlation between predator activity and nymphal burden on Bank voles.

Model	Intercept	Pine marten	Polecat	Red fox	Stone marten	ΔAICc	ω_m
1	**-1.54**					0	0.595
2	**-1.65**	-1.04				2.91	0.139

Table S4.6. Model outcomes for the correlation between predator activity and nymphal burden on Wood mice.

Model	Intercept	Pine marten	Polecat	Red fox	Stone marten	ΔAICc	ω_m
1	-0.54			-1.29		0	0.263
2	-0.38					0.01	0.261
3	-0.49		-1.12			1.43	0.129
4	-0.61		-0.87	-1.17		2.74	0.067
5	-0.39				-0.30	3.04	0.058
6	-0.57			-1.38	-0.49	3.15	0.054
7	-0.38	-0.096				3.23	0.052
8	-0.54	-0.024		-1.29		3.76	0.040
