# Supplementary material for: Cascading effects of predator activity on tick-borne disease risk
Source: Proc Biol Sci. 2017 Jul 19;284(1859):20170453. doi: 10.1098/rspb.2017.0453 (PMC5543215; doi:10.1098/rspb.2017.0453)
Supplement: Supplementary material S5 [file rspb20170453supp5.pdf]

Supplementary material belonging to the manuscript: Hofmeester, T.R., Jansen, P.A., Wijnen, H.J., Coipan, E.C., Fonville, M., Prins, H.H.T., Sprong, H., and van Wieren, S.E. Cascading effects of predator activity on tick-borne disease risk. Proceedings of the Royal Society B: Biological sciences. DOI: 10.1098/rspb.2017.0453

Supplementary material S5.

Full model results of exploratory analyses testing the correlations between rodent characteristics (density, larval and nymphal burden), the density of questing *Ixodes ricinus* nymphs (DON), and the density of infected nymphs (DIN) for *Borrelia afzelii*, *Borrelia miyamotoi* and *Candidatus* Neoehrlichia mikurensis. We did not include nymphal burden in the analyses for Wood mouse (*Apodemus sylvaticus*) as nymphal burden was correlated with larval burden in this species (LMM: $\beta = 0.6$, $p = 0.006$).

Models for DON and DIN were generalized linear mixed models with a negative binomial distribution and log link function with a random intercept per vegetation type nested within year. The average relative humidity measured in the vegetation while drag sampling for ticks was taken into account as a covariate in the models of DON and DIN. As rodent densities, larval burdens and nymphal burdens were over-dispersed, we $\log_{10}$ transformed these parameters to approximate normality. As nymphal burden contained zero estimates, we added the lowest measured positive number (0.01) to circumvent problems with the transformation. Values given are estimates of the standardized correlation coefficient. Significant coefficient estimates are presented in bold, * for $p < 0.05$, ** for $p < 0.01$, *** for $p < 0.001$.

Table S5.1. Model outcomes for the correlation between Bank vole (*Myodes glareolus*) density, larval and nymphal burden with DON and DIN.

Model	Intercept	Log_{10} Density	Log_{10} Larval burden	Log_{10} Nymphal burden	Humidity
DON	3.32	0.23	**1.30*** **	-	0.013
DIN *B. afzelii*	**7.10*** **	-0.17	**1.50*** **	-0.017	**-0.092***
DIN *B. miyamotoi*	3.00	-0.039	**1.20***	0.088	-0.042
DIN *Ca.* N. mikurensis	4.38	0.38	**2.05*** **	0.064	-0.050

Table S5.2. Model outcomes for the correlation between Wood mouse density and larval burden with DON and DIN.

Model	Intercept	Log_{10} Density	Log_{10} Larval burden	Humidity
DON	4.46	-0.023	**1.51*** **	-0.0062
DIN *B. afzelii*	**9.22** **	-0.64	**1.16***	**-0.13** **
DIN *B. miyamotoi*	5.49	-0.37	**0.97***	-0.081
DIN *Ca.* N. mikurensis	**8.50** **	-0.72	**1.82** **	**-0.11***
