# Supplementary figures and images for: Cascading effects of predator activity on tick-borne disease risk
Source: Proc Biol Sci. 2017 Jul 19;284(1859):20170453. doi: 10.1098/rspb.2017.0453 (PMC5543215; doi:10.1098/rspb.2017.0453)

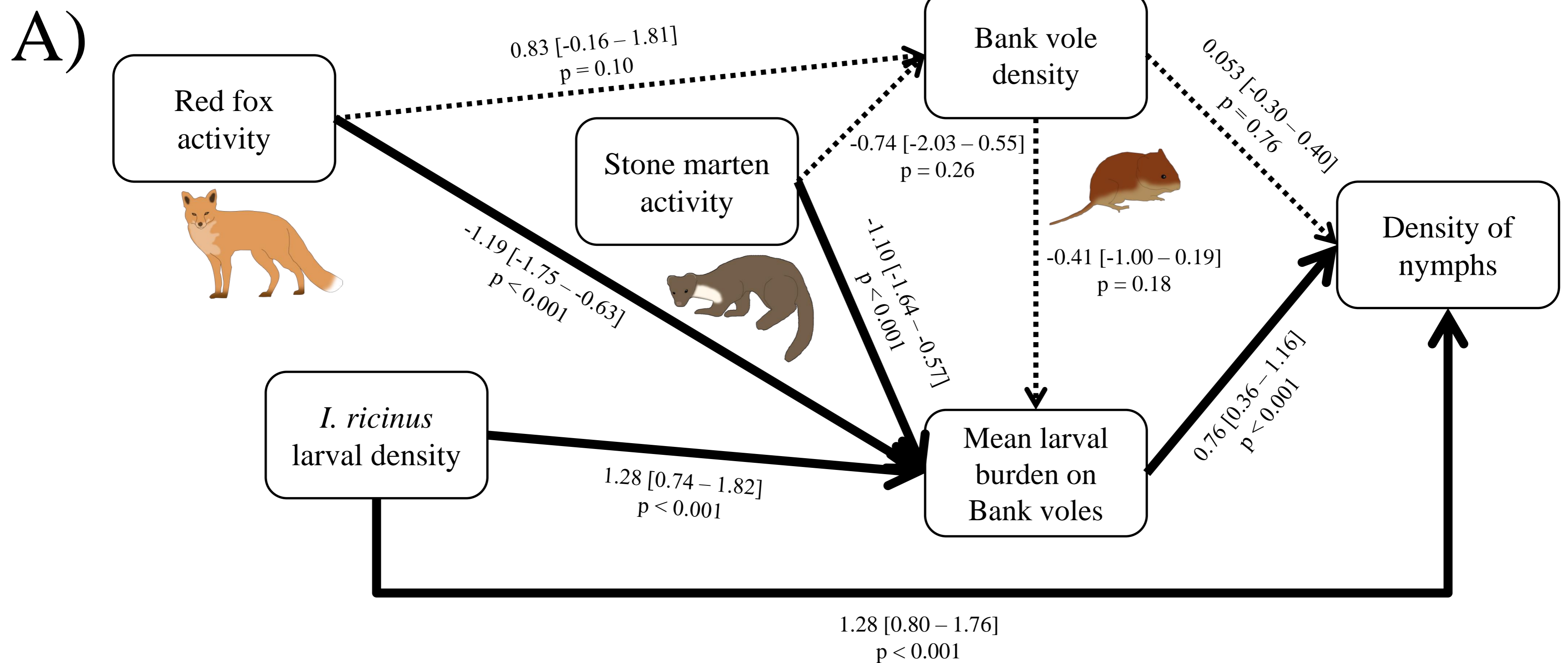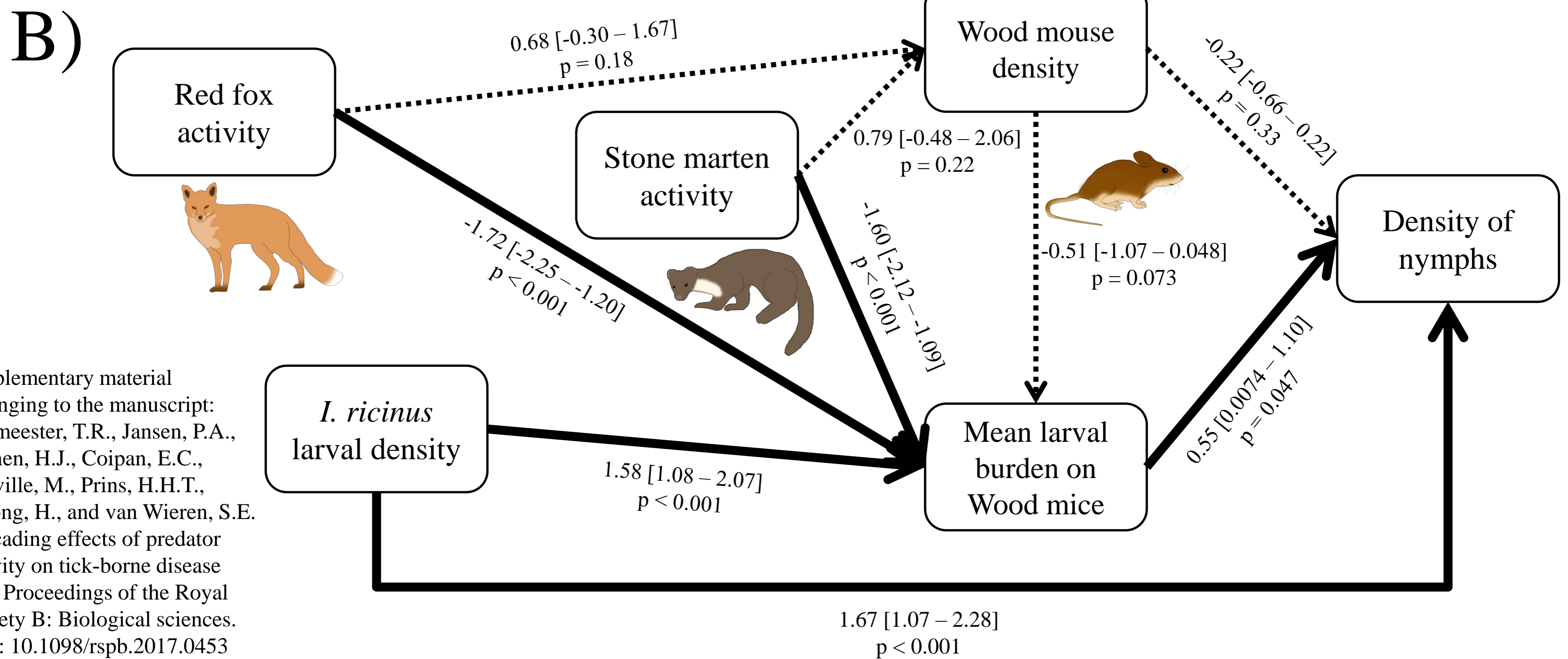

Supplement: Figure S6 [file rspb20170453supp6.pdf]
